# Mobile Zoos and Other Itinerant Animal Handling Events: Current Status and Recommendations for Future Policies

**DOI:** 10.3390/ani13020214

**Published:** 2023-01-06

**Authors:** Clifford Warwick, Anthony Pilny, Catrina Steedman, Tiffani Howell, Albert Martínez-Silvestre, Vanessa Cadenas, Rachel Grant

**Affiliations:** 1 Emergent Disease Foundation, 71-75 Shelton Street, Covent Garden, London WC2H 9JQ, UK; 2 Arizona Exotic Animal Hospital, 2340 E Beardsley Road Ste 100, Phoenix, AZ 85024, USA; 3 School of Psychology and Public Health, La Trobe University, P.O. Box 199, Bendigo, VIC 3552, Australia; 4 Catalonian Reptiles and Amphibians Rescue Centre (CRARC), 08783 Masquefa, Spain; 5 Animal Protection Biodiversity & Environment Section, Government of Catalonia, 43004 Tarragona, Spain; 6 School of Applied Sciences, London South Bank University, 103 Borough Rd, London SE1 0AA, UK

**Keywords:** mobile zoos, mobile live animal programs, animal assisted interventions, animal welfare, public health, safety, injury, one-health, legislation, precautionary principle

## Abstract

**Simple Summary:**

Mobile zoos are events in which non-domesticated (exotic) and domesticated species are transported to different venues for the purposes of education, entertainment, or social and therapeutic assistance. We conducted literature searches and surveyed related government agencies regarding existing provisions within laws and policies, number of mobile zoos, and formal guidance issued concerning operation of such events in 74 countries or regions. We also examined guidance standards for mobile zoos, assessed promotional or educational materials for scientific accuracy, recorded the diversity of species in use, and evaluated those species for their suitability for keeping. We recorded 14 areas of concern regarding animal biology and public health and safety, and 8 areas of false and misleading content in promotional or educational materials. At least 341 species were used for mobile zoos, which are largely unregulated, unmonitored, and uncontrolled, and appear to be increasing. Poor animal welfare, public health and safety, and education raise serious concerns. Using the precautionary principle, we advise that exotic species should not be used for mobile zoos.

**Abstract:**

Mobile zoos are events in which non-domesticated (exotic) and domesticated species are transported to venues such as schools, hospitals, parties, and community centres, for the purposes of education, entertainment, or social and therapeutic assistance. We conducted literature searches and surveyed related government agencies regarding existing provisions within laws and policies, number of mobile zoos, and formal guidance issued concerning operation of such events in 74 countries or regions. We also examined governmental and non-governmental guidance standards for mobile zoos, as well as websites for mobile zoo operations, assessed promotional or educational materials for scientific accuracy, and recorded the diversity of species in use. We used the EMODE (Easy, Moderate, Difficult, or Extreme) algorithm, to evaluate identified species associated with mobile zoos for their suitability for keeping. We recorded 14 areas of concern regarding animal biology and public health and safety, and 8 areas of false and misleading content in promotional or educational materials. We identified at least 341 species used for mobile zoos. Mobile zoos are largely unregulated, unmonitored, and uncontrolled, and appear to be increasing. Issues regarding poor animal welfare, public health and safety, and education raise several serious concerns. Using the precautionary principle when empirical evidence was not available, we advise that exotic species should not be used for mobile zoos and similar itinerant events.

## 1. Introduction

Mobile zoos and other itinerant live animal programs are known by various descriptions, including mobile live animal experiences, animal workshops, animal educational visits, travelling animal shows, animal education events, animal assisted interventions, and others [1,2,3,4]. Mobile zoos, and similar events, share strong commonalities regarding their operational policies and procedures despite differing terminology. Animal assisted interventions are significantly variable, and nine distinct types have recently been named according to different situations targeting mental, emotional, or physical support, with the term ‘visiting/therapeutic animal’ being considered most appropriate for targeted therapeutic events described herein [1].

Essentially, both non-domesticated species (e.g., scorpions, tarantulas, frogs, salamanders, turtles, lizards, snakes, parrots, owls, lemurs, and mongooses) and domesticated species (e.g., dogs, cats, horses, and goats) are transported to venues such as schools, hospitals, parties, and community centres, for the stated purposes of education, entertainment, or social and therapeutic assistance as part of broader-termed ‘mobile live animal experiences’ [1,2,3,4]. Whilst these events may frequently be described and considered collectively, significant differences can be noted in their rationale and operation. Mobile zoos and similar events characteristically or exclusively use non-domesticated wild-animal (also called exotic) species, whereas operations involving visiting/therapeutic animals and similar situations characteristically or exclusively employ domesticated species [5]. Interestingly, the International Association of Human-Animal Interaction Organizations (IAHAIO) guidance considers domesticated visiting/therapeutic animals to constitute ‘partners’ in the assistance effort, potentially implying a mutually amicable arrangement, which is unlikely compatible with the use of wild animals [5]. As a general guide, the terms ‘exotic’ and ‘domesticated’ are valuable [6], but some degree of leniency is required for their use, including in this report, as will be discussed later.

Mobile zoos, in particular where exotic animals are involved, have raised concerns regarding animal welfare, public health and safety, spread of emerging diseases, and miseducation from numerous organisations, which call for greater controls, boycotts, or bans on key activities [2,5,7,8,9,10]. Certain local governments have recently declined requests to add exemptions to their animal control bylaws that would allow the keeping and use of a broad range of otherwise prohibited animals for public display or mobile zoo operations (e.g., [11,12]), and other governments have banned mobile zoos or their activities [13,14].

In contrast, certain animal assisted interventions, especially for therapeutic reasons, are frequently acknowledged for their potentially positive roles, in which species with affiliative or socially adapted histories, such as, domesticated dogs, cats, horses, farm animals, guinea pigs, rats, and birds are involved [5,7,15,16]. Of the species targeted for therapies, domesticated dogs appear to be the primary animals involved [17,18,19,20,21]. Some reports suggest that exotic animals, such as arachnids, amphibians, and reptiles, contribute favourably to therapeutic programmes [c.f. [3]], although those conclusions were based primarily on public responses to novel animals and not on either evidence-based welfare considerations or detailed assessment of zoonotic threats. Reports regarding human observation of aquarium fish have also been reported to have therapeutic values [22], although similar or the same benefits were also noted for people who observe digital screens of moving fish [23,24]. Recorded audio bird song has also been reported for its therapeutic effects [25], and artificial intelligence robots have been successfully used to provide similar benefits to those from visiting/therapeutic animals [26,27]. A recent systematic review found that while there were potential health benefits to people interacting with aquarium fish, research and evidence was limited, with concerns regarding possible historical study biases being cited [28]. Visiting/therapeutic animal programs benefit by involving domesticated species that are adapted to human interaction, with well supported long-standing management protocols, regulations, assurance schemes, and widely available expert veterinary intervention [29,30].

Features of nature, whether plant life, animal life, or habitat scenery, have long been documented as providing interactive health benefits for humans [31,32,33], thus, it is reasonable and desirable for humans to interact with animals in some situations. However, human-other animal interactions should be carefully and not arbitrarily considered. Accordingly, where situations involve intended benefits for participants (and arguably also any true benefits for animals), such benefits should be balanced carefully with potential negative effects, including to the animals used (e.g., housing, transportation, and handling stress) as well as to people (e.g., infections, injuries, and the consequences of miseducation). For example, animal assisted interventions using dogs are well-documented for reducing human anxiety, lowering problematic blood pressure, decreasing related respiratory rates, and improve emotional health (e.g., [34,35,36]). However, some groups, such as hospitalised infants, certain ethnic groups, and other vulnerable patients are at acknowledged increased risk of zoonoses from contact with any assistance animals [36].

In addition, for animal assisted events (and other mobile situations), whilst some animals probably experience positive states, others probably experience negative states. For example, some animals, such as human-familiarised dogs, can display positive engagement with people and experience good welfare within their home environments, transportation, and handling [5]. However, other species, such as snakes and lizards are typically confined to highly restrictive and otherwise inappropriate captive environments, transported under minimalistic conditions, and subject to further handling stress, all of which are associated with captivity stress, morbidity, and mortality [5,37,38,39].

Whilst a substantial number of reports are available regarding animal-assisted therapies, comparatively few reports are available regarding mobile zoos in their various forms. This report will focus primarily on mobile zoo-type events that involve exotic species. The general lack of data available for mobile zoos means that issues related to scale of operations and proportionality of certain practices could not be estimated. Nevertheless, by our adoption of the precautionary principle, as outlined below, we consider that available information sufficiently allows for numerous relevant generalities to be identified and related recommendations to be formed.

Animal interactions with humans are potentially problematic, especially relating to animal welfare and human health and safety, and the aim of this study is to characterise the types of animals used in mobile zoos, and to identify these risks. We will achieve this aim by presenting a brief review of existing provisions within laws, policies, status, scale of operations, and guidance in relation to mobile zoos in Australia, North America, and Europe, as well as providing guidance and recommendations for both formal and informal policy-making, relying on the precautionary principle when empirical evidence was not available.

Throughout this report we adopt the precautionary principle (or precautionary approach), which is frequently applied in situations where there is scientific uncertainty or evidential deficiency, so that presumptive and cautious actions or policies are promoted in order to guide decision-making [40,41]. For example, the precautionary principle has been applied to recognition of animal sentience and welfare [42,43,44,45,46], formulation of positive lists of species that can be traded and kept [40,47,48], biodiversity conservation [49,50], public health protection [41], and is otherwise enshrined in related national and international legislation [40,49].

## 2. Methods

We conducted a literature search using Google Scholar and the following terms for reports published from 2000 (Box 1):

Box 1 **Search Terms for Mobile Zoos**

**Combined with Search Terms for Public Health and Welfare**

**Combined with Terms to Further Refine the Search (- Sign Indicates Exclusion)**
Mobile OR traveling animal experiencesZoonoses, zoonoticExoticMobile OR traveling zoosWelfareWildlifeMobile OR traveling menageriePublic Health-DogMobile OR traveling animal shows
-EquineMobile OR traveling animal exhibit
-CatMobile OR traveling animal encounters
-HorseAnimal assisted intervention OR therapy
-Domestic

Additional items were supplemented from authors’ libraries. Reports were excluded on the basis of low relevance, for example, articles focused on popular history of events or duplication of same information. We also conducted a limited search using the first five pages of Google and approximately 10 items per page for mobile zoos using the single term ‘mobile zoo’. A separate search was performed for businesses offering mobile zoo services in Australia, North America and Spain, The Netherlands, and United Kingdom (English = ‘mobile zoo’; Dutch = ‘dieren huren/dieren verhuren’; Spanish = ‘zoo movil’). Test searches in the UK using the term ‘mobile zoo’ versus the alternative terms on the first page of Google as listed above for the Google Scholar search were also conducted to check for cross-referencing matches for capture of relevant operations.

We examined websites for all mobile zoo operations identified during the limited search using the first five pages of Google and recorded the diversity of species in use. We used the EMODE algorithm [51,52] to evaluate all species that were identified during the searches as being used in mobile zoos, regarding their suitability to be kept captive. EMODE scores animals as ‘Easy’, ‘Moderate’, ‘Difficult’ or ‘Extreme’ to keep according to degrees of husbandry challenge and potential public health and safety risks. The algorithm utilises six pre-weighted closed questions, regarding: 1. species with known sensitivities (e.g., an animal of diminutive physical size that is at risk of handling injuries, or an animal with inherent breed difficulties); 2. species with potentially long lifespans (e.g., an animal that may live 10 years or longer, which presents significant care commitments); 3. species with highly specialised nutritional needs (e.g., an animal for which nutrition can be difficult to obtain); 4. species with needs for specialised habitats (e.g., an animal that is environmentally dependent on a particular rare plant); 5. species that present clear risk of appreciable injury to humans (e.g., an animal that is large, powerful, poisonous, or venomous); and 6. people vulnerable (household-specific) to zoonotic infections (e.g., children under 5 years, the elderly or pregnant, those diagnosed with HIV or other immune diseases, drug users, and those receiving chemotherapy, such as cancer and anti-rejection treatments). Each of the six questions that are affirmed for the relevant species are assigned 5 points, and the combined scores assign the animal to one of the four categories (Easy—Extreme) mentioned previously. The EMODE algorithm has received wide support and promotion, including from animal welfare organisations, the British Government Home Office, local governmental departments, and from within the veterinary profession (e.g., [53,54,55,56]).

We also assessed promotional or educational materials produced by mobile zoo operators for scientific quality and compared information using recent peer-reviewed texts. We contacted government agencies in 74 countries or regions (comprising 6 States in Australia, 50 States in the USA, 9 Provinces in Canada, and 7 European countries) for information regarding existing provisions within laws and policies, number of mobile zoos, and formal guidance issued concerning operation of such events. We evaluated governmental and non-governmental guidance standards for information quality regarding mobile zoos, including matters of animal husbandry and public health and safety. Contacts with government agencies were made through emailed surveys using predetermined questions, which were: 1. Do you have mobile zoos in your jurisdiction? If so, how many? 2. What laws/regulations, if any, do you have regarding mobile zoos? and 3. What guidance, if any, do you provide to regulate mobile zoos?

## 3. Results

A total of 473 peer-reviewed reports were identified, and 121 relevant reports were analysed. The test searches in the UK using the term ‘mobile zoo’ versus the alternative terms listed above for the Google Scholar search resulted in cross-referencing matches of 19 *v* 26 (73%); thus, the term ‘mobile zoo’ was efficient at identifying relevant targets. Searches performed in naturally non-English speaking countries (Spain and The Netherlands) using respective terms for ‘mobile zoo’ located similar average numbers for page listings (i.e., 4 per page). Thus, the common terms used probably located significant examples of relevant events.

Of the 74 countries or regions contacted for information regarding existing provisions within laws and policies, number of mobile zoos, and formal guidance issued concerning operation of such events, 37 survey responses were received from Australia (5 States), USA (26 States), Canada (3 Provinces), and from Belgium, Wales and England, although the information provided was largely incomplete. Supplementary information was located through online searches.

### 3.1. Provisions within Laws and Policies

Identifying consistent laws and policies across local countries or regions regarding mobile zoos and related events was challenging. Much information provided by governments was incomplete, thus Table 1 contains widely varying content. A recent summary of US State laws regarding the exhibition of exotic animals is available elsewhere [57].

### 3.2. Quantifying Mobile Zoos in Australia, North America, and Europe (Spain, The Netherlands, and United Kingdom)

Online searches for businesses offering mobile zoos services listed on the first five pages of Google identified the following numbers: Australia *n* = 25; USA *n* = 25; Canada *n* = 13; Spain *n* = 20; The Netherlands *n* = 17; UK *n* = 19. Only partial information regarding number of mobile zoos and individual events per selected country was established. Very few government agencies contacted could provide information on number of mobile zoos operating in their region, largely because such events are either unregulated or only partially regulated with only certain species requiring permits. In Australia, Queensland, 85 mobile zoos were registered [95]. In Maryland, United States, ten mobile zoo operations are reported across the State that provide educational programs under the oversight of Maryland Park Service [96]. In Pennsylvania there were 88 registered menageries (not necessarily mobile) [97]. Tennessee Captive Wildlife officials report that between 62 and 70 mobile zoos have occurred during the past three years [98], although the report did not specify number of actual operators or events. In Alaska two to five educational permits have been issued for travelling animal exhibits, mostly raptors [99]. In Canada, Quebec, four temporary animal-in-transit permits were issued in 2022 [100]. In The Netherlands, a nongovernmental general advertising registry cites 4800 mobile animal event operators in that country [101]. In the UK, there are reported to be >187 mobile zoos operators using a combined number of 3500 animals [102].

### 3.3. Formal Guidance

Our limited survey of guidance issued by government agencies regarding provisions within laws and policies identified numerous regulatory measures that were in place to alleviate, notably, public health issues relating to mobile zoos and animal assisted therapies. Whilst not a comprehensive review, these examples represent the types of measures currently in place for regulating mobile zoos and visiting/therapeutic animals.

In the United States, guidance typically contains precautions in accordance with the standard measures issued by the Centers for Disease Control and Prevention, which focuses on handwashing [103]. In Australia, New South Wales, there is specific regulation [58] and published guidance [59] for exhibition of animals at mobile establishments. The guidance focuses on animal welfare, but also covers issues concerning public health and safety and educational value of exhibits. Western Australia adopts this same guidance in their ‘Code of practice for exhibited animals in Western Australia’ [67] and in addition their Department of Health issues ‘petting zoo guidelines’ [68] focusing on public health, including advice on disease transmission and hygiene precautions in accordance with the standard measures issued by the Centers for Disease Control and Prevention. In Victoria the ‘Code of Practice for the Public Display of Exhibition of Animals’ [65], and in Queensland the ‘Exhibited Animals Act 2015′ [60], manage the risks associated with animal welfare, biosecurity, and safety. In the United Kingdom, government advice contains the following provisions: all pets in education and childcare settings: animals are always supervised when in contact with students; students and staff are advised to wash their hands immediately after handling animals; animals have recommended treatments and immunisations, are regularly groomed (including claws trimmed) and checked for signs of infection; bedding is laundered regularly; feeding areas are kept clean and their food stored away from human food; food is not consumed within 20 min and is taken away or covered to prevent attracting pests; reptiles are not suitable as pets in education and childcare settings as all species can carry salmonella which can cause serious illness [4].

### 3.4. Species Diversity

Across the six surveyed countries for which relevant information could be obtained a total number of at least 341 taxa (including subspecies) were identified as used for mobile zoo activities, which represented the following: classes and numbers of species for each class: invertebrates *n* = 68; fishes *n* = 15; amphibians *n* = 17; reptiles *n* = 102; birds *n* = 63; mammals *n* = 76. Table 2 provides a further breakdown of animals by class and species involved in mobile zoos for each surveyed country.

### 3.5. Suitability of Species to Keep or Use for Mobile Zoos

Table 3, Table 4, Table 5, Table 6, Table 7 and Table 8 provide lists of animals by class and species that were identified as associated with mobile zoos, as well as the countries in which they were identified. Table 3, Table 4, Table 5, Table 6, Table 7 and Table 8 also include the EMODE primary scores given in points, followed by the challenge determination for all species identified at mobile zoos. Where exact species were not pre-scored online, ‘lookalike’ species were used to ascertain suitability scores (i.e., species of very similar biology and behaviour related to same genus types. However, the scores provided in Table 3, Table 4, Table 5, Table 6, Table 7 and Table 8 have not been adjusted for vulnerable groups, because this question requires household-occupant input. Of all 341 species identified at mobile zoos, the husbandry challenges and numbers of animal types were determined as follows: Easy *n* = 3; Easy—Moderate *n* = 39; Moderate *n* = 20; Moderate–Difficult *n* = 5; Difficult *n* = 161; Difficult–Extreme *n* = 78; Extreme *n* = 35.

### 3.6. Education

Table 9 provides a summary of educational messaging common anecdotal literature associated with mobile zoos and their proponents, which are listed as ‘claims’, together with academic evidence-based responses, which are listed as critical comments. Message advocates have been anonymised to protect identities.

### 3.7. Animal Welfare

Table 10 Provides examples of animal welfare concerns identified in peer-reviewed literature that are relevant to mobile zoo practices, together with example originating sources.

### 3.8. Public Health and Safety

A paucity of data exists regarding recorded cases of zoonoses associated with mobile zoos, animal-assisted therapies, or similar static events such as petting zoos. Whilst mobile zoos specifically may not be implicated in many of these cases of infection, the broadly similar nature of animal interactions across related events may suggest important relevance of case histories. Some examples, although minimal, are available for infections contracted from exotic species and domesticated species at relevant events. In 2004, a review of public health data during 12 years identified approximately 800 human case infections associated with open farms, agricultural fairs, petting zoos, and animal exhibits at childcare centres across Australia, New Zealand, Tasmania, USA, Canada, The Netherlands, England, Wales, and Ireland [167]. In the USA, during 2004–2005, an outbreak of *Escherichia coli* (*E. coli* O157:H7) infection gastroenteritis linked to a petting zoo resulted in 100 cases of disease [168]. Also, in the USA, between 1997 and 2007 at least 17 disease outbreaks affecting over 1300 people were attributable to agricultural farms and petting zoos in relation to *E. coli* infections alone [169]. For the years 2011 to 2013 in Western Australia, South Australia, and Queensland combined, there were five recorded outbreaks involving *Cryptosporium* spp., Shigatoxin-producing *E. coli*, and Salmonella typhimurium associated with petting zoos and an animal nursery that affected 83 people [170,171,172,173]. In Austria, in 2016, seven people were infected with *E. coli* [174].

A range of epidemiologically significant pathogens were identified in the literature as frequently occurring among the species associated with mobile zoos or petting zoos, including: *Campylobacter* spp., *Clostridioides difficile*, *Coxiella burnetti*, *Citrobacter freundii*, *Cryptosporidium* spp., *Escherichia* spp., *Klebsiella* sp., *Listeria monocytogenes, Salmonella* spp., *Shigella* spp., *Staphylococcus aureus*, *Pseudomonas* spp., and *Yersinia enterocolitica* (including antibiotic resistant strains) [127,169,174,175,176,177,178,179,180,181,182,183,184]. Numerous zoonotic parasites have also been identified at animal assisted interventions in Italy, including *Eucoleus aerophilus, Giardia duodenalis, Toxocara canis, Ancylostomatidae* sp., associated variously with equids, dogs, cats, and birds [185].

## 4. Discussion

The online search of the first five pages of Google identified between number of mobile zoo operations (13 to 25) identified via Google per country, state or region, although these data are likely underestimated, because operators are known to promote their activities using methods outside of Google (e.g., Facebook or private websites). For example, in the UK our search may have identified approximately 10% sample size of actual mobile zoo operators, whereas in countries with far larger populations, such as the USA, a search of five pages of Google limits catchment to approximately 50 listings, and thus probably represents a lower proportion of operators. The Netherlands, although not a large country, appears to have a large number of operations, but based on the listing service many of these may be aimed at peripheral activities such as product advertising in which animals are used.

### 4.1. Governmental and Nongovernmental Guidance

Governmental agencies have clear obligations to collate and disseminate objective, impartial, and evidence-based guidance to both businesses and the public. However, such information may not always meet these standards, and instead derive at least in part from unqualified, vested interest, sectors (such as within the pet trading and hobbyist community) and consequently be questionable, misleading, or false [110,186,187,188]. Numerous studies have shown that guidance regarding both non-domesticated and domesticated animal husbandry, including that issued by formal authorities, is frequently not adhered to by recipients or poorly followed [53,112,135,186,187,189,190,191,192,193,194,195]. Similarly, guidance regarding public health and safety protocols is also poorly followed, and several studies emphasise the poor adoption of guidance by the public [187,196,197,198,199,200,201,202,203]. Accordingly, guidance in general as well as its actual effectiveness must be viewed with considerable circumspection (see also Table 9), and in the following sections we outline key areas of animal welfare and public health that, we believe, establish the groundwork for more stringent and government mandatory control of mobile zoos.

### 4.2. Classifying Exotic or Domesticated Species

The term ‘exotic’ (or ‘wild’) is frequently used to differentiate certain groups of species (e.g., invertebrates, fishes, amphibians, reptiles, wild birds, and wild mammals) from domesticated forms (e.g., dogs, cats, and agricultural livestock) [6,110,204,205]. This issue is relevant to mobile zoos because legislation and enforcement, as well as some educational matters, are often defined by categorising animals as exotic or domesticated [6,206,207,208,209].

The biological basis for domestication is highly specific, and few species or animal types (e.g., breeds) may meet the stringent criteria required, which include essential, psychobehavioural affiliative traits, particular social group profiles, and other factors, which enable these species to successfully live among humans [6,204,205,210]. Accordingly, references to genuine domestication, require a guarded approach. Particular animal types (e.g., common companion dogs [*Canis familiaris*]) may be rationalised to constitute a domesticated form.

### 4.3. Animal Welfare

All animals are considered to have key needs that must be met for in order to achieve good welfare, for which certain fundamental principles and provisions are set out in many established guidelines, laws, and practices, such as the following (summarised): The Five Freedoms, 1. freedom from hunger or thirst, 2. freedom from discomfort, 3. freedom from pain, injury, or disease, 4. freedom to express normal behaviour, 5. freedom from fear and distress [211,212]; the Three ‘F’s (freedom, feelings & function), 1. animals should lead natural lives through the development and use of their natural adaptations and capabilities, 2. animals should feel well by being free from prolonged and intense fear, pain, and other negative states, and by experiencing normal pleasures, 3. animals should function well, in the sense of satisfactory health, growth and normal functioning of physiological and behavioural systems [213]; the Five Welfare Needs, 1. need for a suitable environment, 2. need for a suitable diet, 3. need to be able to exhibit normal behaviour patterns, 4. need to be housed with, or apart, from other animals, 5. need to be protected from pain, suffering, injury, and disease [214].

Accordingly, these principles and provisions variously promote either aspirational- or requirement-based conditions for securing limited animal welfare safeguards. However, biological information aimed at addressing particular specialised needs, such as climate-specific thermal conditions, lighting, humidity, as well as specialised dietary, psychological, and behavioural factors (although arguably implicit) are not emphasised. Mobile zoos inherently involve several potentially problematic issues, including: animal handling, transportation, forced confinement, spatial restriction, environments unregulated regarding temperatures, light invasion, humidity, noise disturbance, vibration, enclosure microclimate-microhabitat conditions, and other factors (Table 10 & [5,37,38,39,113,119,150,215,216,217,218]). These issues have important implications regarding biological needs and welfare.

#### 4.3.1. Species Suitability

Contrary to claims by the mobile zoos sector that the species they use are suited for captivity and handling (Table 9), the determinations using EMODE algorithm regarding the suitability of species to keep or use for mobile zoos indicate that significant inherent husbandry challenges are associated with most species. Also, general claims that many exotic species are amenable to, or even enjoy, being handled (e.g., [219,220,221]) should be regarded with caution. It has been argued that handling of, especially non-affiliative, exotic, species has no natural counterpart except during predation [124]. Therefore, many such animals may perceive their handler as a predator that has captured the individual, which would typically be an abnormal and stressful experience.

#### 4.3.2. Biological Considerations, Needs, & Preferences

Exotic, and in particular ectothermic, species are highly dependent on specific environmental conditions for activity and metabolism in order to maintain homeostasis [104,107,222,223]. Such animals also harbour strong innate (ancestral) psychological and behavioural traits [224,225,226], and the physical (including spatial) elements of environments are of greatly increased importance compared with, for example, endothermic birds and mammals, which are more adaptable [107,227,228]. For example, in reptiles, innateness results in frequently extensive spatio-exploratory and other activities, and inherent psychological and behavioural limitations result in these animals not being amenable to recognise invisible barriers, such as vivara glass, whereas birds and mammals will recognise transparent boundaries and avoid contact or injury with them [229,230].

Considerable scientific work has been conducted within zoo, laboratory, and other captive settings demonstrating that animals prefer, and show less stress in, larger and more environmentally enriched conditions, than in smaller and unenriched conditions [231,232,233,234,235,236,237,238]. Spacious and enriched environments are increasingly accepted to be highly important to welfare [123,215,239,240,241,242]. However, even in larger and more environmentally enriched conditions, such as the most progressive and science-led zoos, animals continue to express a range of captivity-stress-related behaviours and experience negative welfare, which has been referred to as ‘controlled deprivation’ [215,243]. Some commentators argue that where captive environments provide for certain natural needs (e.g., sufficient room for basic movement or exercise, appropriate shelter, food and water, and opportunities for reproduction), then spatial limitations do not raise welfare concerns [244,245]. However, other authors have concluded that provision of apparently essential needs and resultant strong growth and reproduction rates, do not assure good welfare (e.g., [114,122,229,246]). Domesticated dogs and cats can be regarded as offering relevant examples, in that even for these highly affiliative and multigenerational selected species, provision of abundant food, water, shelter, and sociality, among other things, does not negate their behavioural drives for exploratory locomotion, as well as novel sensory, social and other inputs. In nature, few or no animals naturally spend their lives in spaces limited to those of commercial vivaria and other cages, which raises several issues.

Research has shown that non-domesticated and multigenerational domesticated animals continue to have strong ancestral innate drives states related to natural large home ranges, expression of hard-wired psychological and behavioural preferences consistent with needs for greater spatial and enriched environments [114,122,246,247,248]. Space is vital to allow for the performance of natural behaviours [246,249,250]. Essentially, even in large enriched zoos, exploratory behaviours persist among animals and require considerable space, indicating that captives are commonly not satisfied with conditions that might superficially provide for all needs—hence zoo specimens typically require forced containment to prevent their escape [215,246]. Indeed, in numerous examples where elementary provisions, as previously listed, are met, many species (including fishes, amphibians, reptiles, birds, and mammals) often express play [251,252,253], which itself often requires increased space.

#### 4.3.3. Handling & Stress

Apparent docility or compliance during handling may not imply absence of stress. For example, studies have shown that Mediterranean tortoises (*Testudo hermanni*) and bearded dragons (*Pogona vitticeps*), which are widely promoted as docility or even affiliative to humans, manifest signs of stress during human handling, which may go unnoticed by many keepers [38,254]. Similarly, blue-tongued skinks (*Tiliqua scincoides*) are commonly regarded as unstressed by environmental disturbances, whereas behavioural studies infer their sensitivity to generalised noise and light invasions, and resultant stress [149]. In addition, a series of tragic events reported in the general media in which claimed docile or tame animals have injured or killed their keepers or others (e.g., see 4.4.2. ‘Injury risk’) indicate that handler perceptions that individual animals are ‘safe’ for close-contact human interaction require some circumspection. Accordingly, claims that handling necessarily results in animals becoming comfortable with such activities cannot be regarded as reliable.

Whilst animals possess an array of physiological, behavioural, and psychological coping strategies for dealing with stress, these strategies are contextualised by type of stressor, for example, environmental deprivation such as drought or hunger [255], social or predatory threats [256,257], and by duration or repetition [258,259]. Thus, animals may cope relatively well with a single stressor event (such as a single sound disturbance or movement), whereas repeated or multiple stressor events (sometimes referred to as ‘microstressors’) may be considered harmful both in the short and long terms, and could play a role in transforming acute stress into chronic stress [258,259]. Basically, a series of microstressors may not allow animals to recover between stressor events and result in cumulative stress, maladaptation, and disease [255,256,260,261,262,263,264,265,266,267,268].

There are some studies regarding targeted socialisation and desensitisation of wild animals to relieve certain potential stressors such as handling. Benign operant conditioning or target training is widely used among zoo professionals in order to familiarise animals with certain procedures such as veterinary treatments [269,270,271], and some experiments with handling exotics (e.g., snakes) concluded that handling helped to alleviate stress responses [272]. Thus, some animals, including exotics, may have reduced negative responses if handling and other mildly invasive stimuli are carefully managed with animal welfare as a centralised theme. Traditional and well-established zoos have trained individuals who carry out the positive reinforcement training, and it is unlikely that mobile zoos have such resources. However, as indicated earlier, handling in general is recognised as a significant stressor for wild animals and indeed features as a specific method for stressing individuals used for physiological research; thus, its direct role as a stressor is universally acknowledged.

### 4.4. Public Health and Safety

Several well-understood public health and safety issues are relevant to mobile zoos, notably risks regarding: zoonotic infections, allergic reactions, and injuries. Generally, zoonoses refers to diseases that are transmitted from animals to humans [273,274]. At least 200 zoonoses are known spanning all major pathogens classes, which including bacteria, viruses, parasites, fungi, and prions [127,274,275,276,277]. Whilst much is understood regarding the diversity, history, and treatment of zoonotic diseases, relatively little is known about incidence and prevalence, largely because zoonoses frequently superficially resemble regular morbidities (although often more severe and enduring) and thus may not be properly ascertained or recorded [278]. Nevertheless, 61% of human diseases are potentially of zoonotic origin [279] and 75% of global emerging human diseases may be linked to wild animals [275]. Of the known zoonoses, at least 60 are associated with exotic pet species [127,274], which also constitute the majority of species represented at mobile zoos. Frequently listed exotic animal zoonoses include: salmonellosis, *E. coli* infection, campylobacteriosis, leptospirosis, chlamydiosis, vibriosis, lyme disease, bartonellosis, toxocariasis, giardiasis, mycobacteriosis (tuberculosis), Q-fever, cryptosporidiosis, helminthiasis, ringworm, allergic alveolitis, lymphocytic choriomeningitis virus, and leishmaniasis [127,152,274].

Research has also revealed that many animals, for example reptiles, are potential reservoirs for several antibiotic-resistant bacteria [280,281]. Currently, antimicrobial resistance (AMR) is a global challenge in epidemiology, for example, the World Health Organisations has declared AMR to be one of the top 10 public health threats facing humanity [282], and required urgent multisectoral action in order to achieve the Sustainable Development Goals [283]. Mobile zoos and other animal handling events have been identified as constituting particular risks for transmission of zoonotic pathogens. Disease outbreaks associated with regular petting zoos can be more easily tracked due to the static nature of their operation compared with itinerant mobile zoos, and numerous cases have been identified.

#### 4.4.1. Zoonotic Risk

The proportionality of threat from zoonoses caused by exotic versus domesticated species raises various considerations. Exotic species harbour a substantial diversity of atypical pathogens [127], for which potential epidemic and pandemic implications are unclear yet concerning [284]. Exotic species notoriously derive from sources where both the health states and origins of animals is highly uncertain [37,284,285]. We found that at least 341 exotic animal species were in use by mobile zoos, and this diversity of species, source origins, and management histories also infers both significant natural pathogen diversity as well as artificial cross-contamination involving potentially pathogenic microbes at multi-stage holding sites and during transportation [37,285]. Over 13,000 exotic species are involved in the pet trading and keeping sector [206], and most of these are accessible for mobile zoos due to their availability via commercial suppliers that operate in the public domain, thus, potentially increasing all pathogen diversity issues. The species of exotic animals used for mobile zoos are mostly the same as those present in the pet trade and hobby sectors and share similar sourcing histories and zoonoses [37,127,206]. Therefore, it should be presumed that all relevant pathogens identified in the diversity of species in pet trading and keeping also hold parallel significance to the species involved in mobile zoos.

In contrast, domesticated species, such as dogs, are typically sourced via known suppliers and routes, and almost all are captive-bred [286], thus their health and pathogen-type histories are well-understood. Regardless, strong regulatory measures are in place concerning quarantine controls, passports, and permissions for sourcing and supply.

In addition, the objective literature widely guards against handling or keeping exotic species, notably all reptiles, due to disproportionate threats from naturally endemic (commensal) salmonella pathogenesis (e.g., [287,288,289,290]). The proportion of vulnerable groups (e.g., to salmonella infections) in the general population is high [291], inferring strong probabilities that mobile zoo operations aimed at communal centres and social events, such as schools, hospitals, and parties inherently import significant disproportionate risks to public health. Importantly, regardless of messaging, members of the public likely remain naïve to actual transmission risks [201,292]. For domesticated species, potential pathogens harboured as well as associated public health risks are well understood. Relatedly, veterinary training is routinely superior in respect of identifying and educating on zoonoses associated with domesticated species, such as dogs, cats, sheep, cattle, horses and others, and such expertise is also locally and easily available. In comparison, for exotics, such education, expertise, and availability are minimal [293].

#### 4.4.2. Injury Risk

Human injuries from bites, envenomation, stings, or constriction constitute a relatively small yet medically important and problematic concern [136,137,294]. Limited studies in Germany and the United Kingdom have identified several hundred relevant incidents involving hospitalisation since 2003 [136,137,139,295]. Examples of serious injury are venomous bites and stings from invertebrates and snakes, bites from large lizards, and constrictions by large boas and pythons [136]. A study of hospitalised casualties due to bites, envenomation, stings, or constriction by exotic animals in the UK found that during six years a total of 760 episodes, 709 admissions, and 2121 days of treatment were recorded [136]. Another UK study using data for 12 years from the National Poisons Information Service identified 321 bites from exotic snakes, involving 300 patients, and 68 species [137]. Whilst case numbers are modest, medical treatment is typically more complex [136,137,296]. The presence of strong, intact, innate defensive and aggressive behaviours, behavioural unpredictability, involvement of atypical potential pathogens, and respective increased treatment demands associated with these animals imply disproportionate risks to public health and safety compared with domesticated species [127].

As provided in Table 3, Table 4, Table 5, Table 6, Table 7 and Table 8, mobile zoos commonly involve a large number of essentially wild venomous, or otherwise toxic, species as well as large predators or other physically dangerous animals, across all classes; with many examples reflected by their high EMODE scores. Whether or not these potentially dangerous animals are perceived or claimed to be docile or long-term captives, tragic animal-human incidences occur regularly, and can be illustrated using the example of large constricting snakes. Fatal human incidents by captive moderate-sized (e.g., approximately two meters) or larger-sized constricting snakes are recorded in the media and elsewhere [294,297,298,299,300,301]. Human casualties of large constricting snakes, even those for which they were confident of docility, are typically subject to sudden attacks and collapse [302]. Accordingly, snake attacks can occur without notice, and cause rapid unconsciousness and death where moderate- or larger-sized animals are concerned, and many venomous or large and powerful species similar present latent risks of injury or death to humans. Allergic reactions from direct contact with animals’ bodies, enzymes, excrement, quills, urticating (stinging) hairs, stings, bites, or envenomation are also increasingly reported across all classes of invertebrates, fishes, amphibians, reptiles, birds, and mammals [129,130,131,132,133,134,135,152]. Whilst individual operators of mobile zoos have promoted their animals as having been surgically ‘devenomised’ [303], predatory attacks can still occur.

In terms of scale of potential physical threat, in the United Kingdom there are, for example, many more dogs (approximately 12–13 million) than exotics (approximately 2 million, including all amphibians, reptiles, birds, and ‘unusual mammals’ combined) [304,305]. There are a large number of fishes, although these pose little physical threat not least because they are rarely physically handled. Almost all exotics are confined to enclosures, of which many or most are effectively impermeable, and are far less frequently touched than dogs, which typically interact openly and very frequently with people. Thus, opportunities for aggressive events and outcomes are predictably far greater between dogs and people. Indeed, due to the popularity of dogs and their closeness to people in the home, there are far more bites associated with dogs [306,307] than there are known from exotics [136].

#### 4.4.3. Infection Control

Available government and other guidance for infection control at mobile zoos typically emphasises post animal contact handwashing as well as cautions when eating or drinking around novel animals, (e.g., [4,70,308,309]). However, whilst normal handwashing is a useful method for reducing microbes [310,311], it is not a comprehensive measure against pathogen contamination [125,126,201]. There are various reasons for the inadequacies of handwashing and other hygiene measures in safeguarding health. For example, a study comparing alcohol, ozonized water, and soap and water found that eradication of *Escherichia coli* was effective in 10 out of 35 participants, 10 out of 55 participants, and 6 out of 20 participants, respectively [126]. A systematic review of studies regarding the effectiveness handwashing in controlling respiratory and gastrointestinal infections among children in educational settings found that evidence was equivocal, nevertheless handwashing should not be deterred [128].

Studies of handwashing and other hygiene protocols amongst medical staff, including at intensive care units, in which infection control is a heightened concern, was found to be variable, but overall poor and involve low levels of adherence to best practices [312,313]. It is estimated that hospital acquired infections generally in the UK may affect as many as 23% of admissions [314], and result in the deaths of approximately 5000 people per year in England [315]. Studies of zoonotic episodes among veterinary professionals reported that approximately between 16% [316] and 20% [317] of staff experienced zoonotic disease during five years, and whilst veterinarians confront large numbers of animals of uncertain backgrounds, disease prevention is clearly unsuccessful regardless of greater than average awareness of zoonoses in the sector. Therefore, even where mandated and performed by highly professional medics who understand the importance of microbial decontamination, disease prevention and control measures remain incomplete and present a significant risk to public health. Accordingly, handwashing, as a common recommendation, can be useful in reducing disease if conscientiously performed, but has important weaknesses and is subject to over-reliance and may invite complacency.

At animal contact events, general contact behaviours are likely to result in rapid recontamination of even cleaned hands from microbes dispersed prior to washing (e.g., hands touching clothes and recontaminating washed hands), largely negating any sanitisation advice or practices [34,201,202], with significant implications for petting zoos and mobile zoos [197,200]. Relatedly, infections continue unabated at mobile zoos and related events regardless of handwashing measures [70]. Therefore, regular infections at mobile zoos are arguably highly predictable considering the inherent biohazard of exotic animals and related pathogens.

Approximately 14% of all infections from *Campylobacter* spp., *Cryptosporidium* spp., *Escherichia* spp., *Listeria monocytogenes, Salmonella* spp., and *Yersinia enterocolitica* are estimated to arise from animal contact alone [318]. Whilst the potential representation of these bacteria at mobile zoos versus society in general appears not to have been researched, the presence of these prevalent and important pathogens at such events is important to note. The persistence of these bacteria in normally highly controlled clinical settings as well as at mobile zoos, indicates that not only do these pathogens frequently evade even high-level hygiene practices, but also would likely be masked as HCAIs among presenting hospitalised patients, who may in fact have acquired infection from contact with visiting animals [278,296]. Considering the large volumes of people exposed to exotic animals at mobile zoos, and accounting for further reduced hygiene practices at such itinerant events, infection risk is clearly more significant than among clinical environments.

As reported previously, some guidance issued by relevant ‘thinktank’ non-governmental organisations and academic researchers recommends against the use of exotic species in assisted therapy contexts, due to zoonotic risk factors and difficulties of pathogens control (e.g., [5,7,197,319,320]). Such precautionary guidance is accepted for constituting efficient and economical prevention and control of case infection and epidemics [175,277,321]. these guidelines are efficient but not mandatory. Therefore, it is difficult to establish non-governmental protocols to prevent and control diseases. Such guidelines may be efficient, but their use may not be mandatory. Therefore, it is difficult to establish non-governmental protocols to prevent and control diseases.

#### 4.4.4. Epidemiology and Surveillance

Establishing or estimating the incidence or prevalence of infections linked to mobile zoos is confounded by several well-known factors. Many zoonoses superficially present as common infections, such as gastrointestinal, flu-like, and dermal diseases; albeit that zoonotic episodes often manifest as more severe or persistent forms [34,127,322,323]. Patients of zoonoses acquired from mobile zoos may experience diagnostic lag-phases associated with delayed onset of disease; thus, they may fail to link their illness to visiting live animal handling experiences. Doctors and other healthcare professionals may not ask relevant questions of presenting patients regarding possible animal contact histories, despite strong and repeated recommendations to do so [278,296,309,323,324,325]. Even if correctly diagnosed, trace-back may then present difficulties in affirming a precise location and cause of the infection, due to the itinerant displays and because species and individuals used by mobile zoos are frequently changed [326]. An allied issue of growing concern is the frequently minimal management of residual waste associated with zoonotic cases, which can have potential to initiate some epidemic outbreaks [327].

### 4.5. One-Health, One-Welfare

The terms ‘one health’ and ‘one-welfare’ are co-relevant paradigms linking environment, animals, and people, implying that negative effects in one part of this complex may be transferred to another, warranting multi-disciplinary resolutions [328,329,330]. Poor animal husbandry, stress, and other factors, are directly relevant to the one-health, one-welfare paradigm. As indicated previously, sourcing, supply, and keeping of exotic species, whether for mobile zoos or other sectors, are known to commonly harbour a diversity of factors related to both poor welfare and poor hygiene, including: unknown country of wild-capture, known country of wild-capture being associated with zoonotic hotspots, stressful and unhygienic conditions of captive breeding, stressful and unhygienic conditions of storage, stressful and unhygienic conditions of husbandry, poor veterinary management, high levels of infectious morbidity, high levels of injury, and high levels of mortality [37]. The great diversity of species used for mobile zoos also implies wide variation in biological needs (see 4.3.2. ‘Biological considerations, needs, & preferences’), and this diversity of needs infers corresponding high husbandry demands.

### 4.6. Education and Miseducation at Mobile Zoos

As summarised in Table 9, false and misleading claims regarding animal biology, husbandry, and public health and safety were commonly identified via mobile zoos websites, promotional materials, and presentation messaging, although we did not calculate the frequently of such information by percentage of representation. Regarding animal biology and husbandry issues, the standard of information and apparent knowledge was considered to be poor and consistent with what is broadly referred to as ‘folklore’ or ‘arbitrary’ husbandry which is frequently based on handed-down, outdated, unproven, inaccurate, misleading or dangerous information [104,105,331]. Such information inadequacies frequently involve negative animal welfare implications [104,105]. Whilst some miseducational content could potentially be corrected by input of objective evidence-based information from bona-fide impartial experts, such material would likely be ignored where it contradicts and disfavours regular mobile zoo promotional messaging [114]. Relevant examples include claims that animal welfare is safeguarded at itinerant events, which would instead require re-messaging that would necessarily state that animals used likely experience stress, and that apparent behaviours do not indicate quiescence or suitability for handling [219,221]. Also, broader biological facts would also need to reflect that captive-breeding of animals does not indicate domestication or their suitability for keeping or handling [6].

Regarding hygiene and other disease transmission issues, as well as injury risk prevention, the standard of information and apparent knowledge was again considered to be poor based on accepted peer-reviewed public health guidance information [127], and to be improved would need to convey alternative messaging that no regular measures, such as handwashing, can be considered protective, and that all animals (especially non-domesticated species) present significant threats to public health and physical safety, regardless of background. Claims that furless and featherless animals, such as reptiles, are especially safe for handling by people with allergies, which were common at mobile zoo presentations, invite serious risk of complacency with major implications for ill-health.

Importantly, even if objective information was universally mandated and accepted by mobile zoo advocates and followed by attendees at events, such information would not prevent animal welfare and public health and safety problems inherent to mobile zoos, because it would unlikely translate into dependable outcomes [127,187,194,312,313,332]. Such messaging regarding biology and husbandry would not alleviate applied stressors and other negative impacts inherent to mobile zoos, such as transportation, temporary holding sites, and contact or handling (see 4.3.2. ‘Biological considerations, needs, & preferences’). Enforced handwashing would not reduce microbial loads carried by animals or prevent risk [127]. Selection of only docile species would not eliminate innate defensive or aggressive behaviours among animals in response to perceived threats, and associated injury risk.

### 4.7. Mobile Zoos Versus Traditional Zoos and Static Zoos

Traditional static zoos attract some criticisms on both animal welfare and public health and safety grounds, which are based largely around issues of spatial restriction, lack of environmental enrichment, deficient or problematic social groupings, general captivity-associated stressors and stress, and hygiene concerns [127,201,215,333,334,335]. However, traditional static zoos frequently acknowledge these problems and, whilst potentially not fully resolvable, increasingly adopt formal strategies, undertake dedicated scientific research, cross-share and peer-review operational information via conferences and specific publications, and employ qualified veterinarians and special animal welfare personnel in order to alleviate a range of challenges [104,239,336,337,338,339]. Also, traditional static zoos are regulated in several world regions, requiring inspection and certification, and monitored for management practices (e.g., [207,340,341,342]), although these controls are not without criticism for failing to assure welfare and other concerns (e.g., [343,344]). In contrast, none of these safeguards apply to mobile zoos.

Animals at traditional static zoos are typically not subject to frequent handling (especially by novice members of the public), whereas in mobile zoos they are frequently handled. At traditional zoos, transportation is minimal, and animals are proportionately better insulated against human disturbances associated with sound, vibration, light, smell, and visual confrontation than animals at mobile zoos, which strongly expose animals to all such disturbances. These disturbances are now well-known to impose significant stressors of animals, including formerly poorly understood species, such as reptiles [5,38,119,150,215,254]. Issues of disturbance to animals and reduced abilities to attain homeostasis are negatively compounded where nocturnalism is part of species natural biology, as is commonly the case in many species, and results in animals being handled or transported during their normal rest periods [114,159]. Significantly, for nocturnal species, welfare assessments cannot usually be well performed, because their activity patterns and behaviours signalling health states are not observed due to the contrary diurnal behaviour patterns of humans [152].

Traditional static zoos have been associated with a number of zoonotic outbreaks [345,346], including relatively large episodes involving hundreds of people from a single reptile exhibit [347]. However, infection risks at traditional static zoos can be strongly mitigated in part due to the established architectural layout and thus the predictability of circumstances and events. Most zoos also have biosecurity policies, especially in relation to notifiable diseases (e.g., [207,348]). Hygiene control for public interactions with animals at traditional zoos has also been shown to be over twice as effective than for mobile events [201]. Therefore, the risk for zoonoses at mobile zoos is elevated. In contrast, mobile zoos occur at diverse offsite locations that are significantly beyond public health and safety management predictability, and therefore present a disproportionately great risk of both zoonotic disease and (where potentially dangerous animals such as large species of animal are involved) human injury.

### 4.8. Control Measures

Various principles are used as measures of control to regulate activities involving humans and animals. The most effective ‘gold-standard’ control approach is to prohibit or ban relevant activities [40,192,246,285,349,350,351]. An alternative and permissive approach is to allow activities that have been independently and scientific demonstrated in advance to present no unreasonable risk to animal welfare, public health and safety, or the environment by including such proven operations on a positive list [40,208]. Positive lists are integral provisions to normal management of risks affecting society, and apply to all major professions and products. Positive lists could theoretically be applied to the employment of, for example, dogs for animal assisted therapies, in that there is good local expert veterinary care available to assess issues regarding animal health and welfare states, husbandry and transportation conditions, and zoonotic risks. However, where exotic animals are concerned, both species and pathogen diversity infer vastly different abilities to ascertain those same issues, and it is highly unlikely that exotic species would meet acceptable criteria for inclusion on positive lists.

## 5. Limitations of Study

Searches during this study for mobile zoos and related operations for each targeted country were limited to the first five pages of Google; thus, capture of a representative sample is uncertain. Relatedly, ascertaining or estimating the number of mobile zoo operators regionally or globally was not feasible. Also, whilst there were strong commonalities between species used for mobile zoos across various regions or countries, some variation was noted, thus the list of species herein may be considered a partial compilation. For example, whilst our survey identified 13 mobile zoos operating in Canada, anecdotal reporting indicates that the actual number is considerably greater [352]. Similarly, whilst a wide range of birds and mammals were identified across surveyed countries, numerous species including, sloths, bobcats, ring-tailed lemurs, and reindeers, are anecdotally reported as occurring at Canadian mobile zoos by observers, despite not being recorded during the limited survey [11,352].

Minimal or absent regional and global monitoring or control of mobile zoos causes large gaps in information regarding scale that could not be determined. Lack of available data regarding confirmed cases of disease associated with mobile zoos and similar activities prevents detailed projections regarding epidemiological risk.

## 6. Conclusions

Our survey of provisions within laws and policies indicated that mobile zoos are largely unregulated, unmonitored, uncontrolled globally, and appear to be increasing in scale. Existing provisions laws and policies are few, mostly under-developed, require urgent reform, lag behind some modern scientific approaches to both safeguarding animal welfare and public health and safety messaging, fail to adequately control the raft of problematic issues inherent to mobile zoos, and require urgent reform. Similarly, governmental guidance in general for managing mobile zoos is minimal and deficient, in particular due to reliance on minimalist and arbitrary husbandry practices and overemphasis on handwashing and public compliance, which invites risk complacency. Our investigation found that educational messaging by mobile zoo proponents was highly variable and frequently false or misleading, and this deficiency raises fundamental questions regarding the supposed role of mobile zoos as information, or misinformation, providers to the general public.

As presented in Section 4.3. (*Animal welfare*), Section 4.3.1. (Species suitability) and Section 4.3.2. (Biological considerations, needs, & preferences), whilst all the animal welfare, public health and safety, and educational concerns discussed previously are relevant to other situations in which handling occurs, such as static petting zoos and animal assisted therapies, mobile zoos, in our view, raise several serious concerns because the animals involved are subject to frequent transportation and associated manipulation. Such transportation and manipulation are likely to induce a series of cumulative disturbance-related microstress episodes that inhibit rest and recovery periods, and promote chronic stress and compromised welfare. Relatedly, chronic stress and poor welfare in animals potentially increase risks of acquired disease, carrier status, and pathogen-shedding, with zoonotic implications recognised by the one-health principle.

There is no formal methodological monitoring for case infections or epidemic outbreaks linked to mobile zoos, despite there being clear evidence of such associations, and the likely attendance of significant proportions of immunologically vulnerable groups. This lack of monitoring is concerning given the prevalence of key pathogens that are both common in society and known to be linked to mobile zoos. As presented in Section 4.4.3. (Infection control), salutary lessons ought to be learned from the persistent healthcare-associated infections occurring in the medical profession, which direct that good hygiene at mobile zoos and related events should rationally be considered unachievable. Relatedly, the lack of recorded cases and outbreaks cannot be interpreted to indicate low prevalence of mobile zoo-associated zoonoses, and although there is likely under-reporting of infections.

As presented in Section 4.1. (*Governmental and nongovernmental guidance*) and Section 4.6. (*Education and miseducation at mobile zoos)*, the uptake of high-quality objective guidance, even in highly regulated and professional sectors including highly regarded zoological institutions and in medicine and surgery, as well as for privately kept animals, is known to be subject to significant inertia and applied difficulty. Therefore, it is probably overly optimistic to presume that (even if improved and mandatory) governmental guidance in respect of animal welfare or public health and safety for operating mobile zoos, or the messaging by operators of these events, can be relied on to meaningfully filter into actual practices or achieve desired benefits, especially where exotic species are involved.

Our evaluations using the EMODE system concur with previous reports that exotic species are not suitable for inclusion in mobile zoo and other similar live animal programs. Accordingly, the use of exotic species at mobile zoos and other handling events infers disproportionate risks to animal welfare and public health and safety. Relatedly, as presented in Section 4.8. (*Control measures)*, we agree that prohibitions on certain practices provide the most secure and reliable method for control and prevention of major areas of concerns regarding mobile zoos. On the basis of the precautionary principle as described earlier, we have developed several recommendations for the control and monitoring of mobile zoos and similar live animal programs.

## 7. Recommendations

1. Exotic (non-domesticated) species, as well as large and potentially physically dangerous domesticated species, should not be used for the purposes of mobile zoos, petting zoos, animal assisted therapies, or any other mobile live animal program. This recommendation is to better protect animals against welfare problems that are associated with the frequently highly specialised biological needs and sensitivities associated with captive wildlife, and to public health and safety from atypical zoonoses and injuries.
2. Animals used for the purposes of mobile live animal programs, should be limited to species that are highly adaptable to and suitable for human interaction, such as amenable individuals of certain types of domesticated dog.
3. All mobile zoos, petting zoos, animal assisted therapies, or any other mobile live animal program operations, should be subject to government mandatory registration and frequent inspection by veterinary or other independent qualified personnel to assess health and welfare states, long-term and short-term or otherwise temporary accommodations, transportation protocols, and operator knowledge.
4. All cases or epidemiological outbreaks of disease at or associated with any mobile zoos, petting zoos, animal assisted therapies, or any other mobile live animal program should be subject to government mandatory notification to regional and national public health authorities.
5. Health and carrier-state screening of all animals, including faecal analysis, for potential pathogens, should be performed frequently to target common relevant zoonotic bacteria and parasites.
6. Formal surveillance of patients at both primary and secondary care interfaces should be increased to target relevant pathogens with overlapping zoonotic histories.

## Figures and Tables

**Table 1:** Provisions within laws and policies for managing mobile zoos by country, state or region.

**Australia** No Specific Federal Government Regulation		
*State*	*Provisions within Laws or Policy*	*Source*
New South Wales	Specific legislation and licensing conditions.	[58,59]
Queensland	Exotic species require exhibition licences, and are covered by specific legislation (which applies to risks to animal welfare, biosecurity and safety) although domestic petting farms are exempt.	[60,61]
South Australia	All zoos are subject to specific permits for displaying native wildlife, although only certain native species require licence. Movement of livestock subject to regulation for biosecurity reasons.	[62,63]
Victoria	Only certain species require licence; includes guidance principles for animal welfare and public health and safety. Authorised officers enforce the POCTA Act and Regulations, and advise people requiring assistance in the operation of mobile zoos.	[64,65]
Western Australia	No licences are required to operate mobile zoos, although these events are required to comply with the Animal Welfare Act (2002), and associated regulations. Specific guidance via ‘Code of practice for exhibited animals in Western Australia 2003′ and ‘Petting Zoo Guidelines’ published by Environmental Health Resource (public health and safety measures).	[66,67,68]
**United States of America** Federal Animal Welfare Act (1966) [69] requires permits for public exhibition of animals. Invertebrates, fishes, amphibians, reptiles, and farm animals are not covered. Birds are covered, although there are no regulatory standards included. Individual States may adopt their own prohibitions and regulations. Many regional departments of wildlife (or equivalents) enforce regulations on keeping or exhibiting native wildlife and interstate movement of animals is often subject to animal health regulations (usually livestock).		
*State*	*Law or policy*	*Source*
New York, North Carolina, Wisconsin	Hand washing requirements.	[70]
Alaska	Educational live exhibition permit required. 2–5 registered mobile exhibitors.	[71]
California	No specific license for mobile zoos but exhibition permit required for species on an approved list.	[72]
Florida	Licence required for specific wildlife only—subject to specific regulations; caging requirements and time limitation on smaller travel caging, itinerary of planned exhibition times and locations.	[73,74]
Michigan	Exhibition requirements for certain species (e.g., cervids, large carnivores, farmed animals) native wildlife or exotic, circus and zoo animals.	[75]
Minnesota	Exhibition of Wildlife permit required and related regulations. Exemption for privately owned traveling zoo or circus.	[76]
Montana	Permit required for wild animal menageries, sanctuaries and zoos. Temporary Exhibitors Permits required for mobile zoos.	[77,78]
Nebraska	Permit required for certain animals in captivity.	[79]
New York	Wild Animal Exhibition Permit. License individuals who travel with animals for education and exhibition purposes but same type of licence for static zoos, thus no numbers, certain conditions attached to license.	[80]
Pennsylvania	Permit required for all ‘wildlife menageries’. Regulations include public safety, humane care, and treatment, adequate housing and nutrition, sanitation, safety, acquisition and disposal of wildlife and exotic wildlife, many species-specific regulations for mammals and birds (e.g., cage sizing).	[81,82]
Rhode Island	Permit required for possession of certain exotic species.	[83]
Tennessee	Regulations and permissions vary according to species, and whether exhibition is for profit. Department of Agriculture also regulates some species.	[84,85]
Texas	No specific mobile zoo regulations.Educational Display Permits required for protected wildlife.Permit required to possess certain species (e.g., non-indigenous snakes).	[86,87]
**Canada** No specific federal government regulation.		
Province	Law or policy	Source
Ontario	PAWS Act—standards of care and prohibitions on causing or permitting distress to an animal. No specific mobile zoo legislation. Some municipalities and public health units in Ontario has by-laws or guidance that may outline requirements or recommendations for mobile zoos at the local level. For example, the Halton Region Health Department provides guidelines for petting zoos, including traveling attractions.	[88,89]
Quebec	Permits required for traveling exhibitions of wild or exotic animals to the public.Permits issued in respect of protecting animal welfare and conservation of wildlife.	[90,91]
Saskatchewan	No specific mobile zoo regulations. Possession of specific species regulated but many species on the ‘allowed’ list (e.g., over 200 species of reptile vs. 11 species of mammal).	[92]
**Europe** No specific EU legislation		
*Country*	*Law or policy*	*Source*
Belgium (Flanders)	Animal Welfare service legislates zoos—physical contact between visitors and animals is prohibited. Travelling exhibitions/mobile zoos are regulated but none at present.	[93]
Ukraine	Mobile zoos banned on animal welfare grounds.	[14]
United Kingdom (England, Ireland, and Wales)	Licences issued under specific regulations. Additionally, new proposals to regulate or license mobile zoos in a similar or same way as used for traditional static zoos.	[2,4,94]

**Table 2:** Numbers of species by class for each surveyed country.

Country	Animal Class	Number of Species
Australia	Invertebrates	36
	Fishes	6
	Amphibians	7
	Reptiles	24
	Birds	15
	Mammals	33
		Total 121
USA	Invertebrates	10
	Fishes	8
	Amphibians	3
	Reptiles	34
	Birds	30
	Mammals	46
		Total 129
Canada	Invertebrates	3
	Fishes	0
	Amphibians	1
	Reptiles	29
	Birds	2
	Mammals	13
		Total 48
Spain	Invertebrates	6
	Fishes	0
	Amphibians	0
	Reptiles	17
	Birds	18
	Mammals	17
	Invertebrates	Total 58
The Netherlands	Invertebrates	3
	Fishes	0
	Amphibians	2
	Reptiles	14
	Birds	5
	Mammals	16
		Total 40
United Kingdom	Invertebrates	32
	Fishes	2
	Amphibians	10
	Reptiles	51
	Birds	22
	Mammals	24
		Total 141
Combined number of species across all surveyed countries		Total 341

**Table 3:** Invertebrates involved in handling and other practices at mobile zoos by species and country of where used, and their EMODE* ‘suitability to keep’ scores.

Species		Country	EMODE Score/Challenge
Scientific Name	Common Name		
*Aurelia aurita*	Moon jellyfish	USA, UK	15 = Moderate
*Octopoda* sp.	Octopus	AUS	28 = Difficult-Extreme
*Crustacea* sp.	Crustacean	AUS	25 = Difficult
*Cherax destructor*	Yabby	AUS	25 = Difficult
*Brachyura* sp.	Crab	AUS	25 = Difficult
*Pagaroidea* sp.	Hermit crab	AUS, UK	25 = Difficult
*Asteroidae* sp.	Sea star	AUS	25 = Difficult
*Liparidae* sp.	Sea snail	AUS	10 = Easy-Moderate
*Mollusca* sp.	Mollusc	AUS	10 = Easy-Moderate
*Lissachatina fulica*	Giant African land snail	UK	10 = Easy-Moderate
*Archachatina marginata*	West African land Snail	NL, UK	10 = Easy-Moderate
*Achatina achatina*	Ghanaian tiger land Snail	UK	10 = Easy-Moderate
*Achatina fulica*	Snail	ESP	10 = Easy-Moderate
*Triboniophorus graeffei*	Red triangle slug	AUS	10 = Easy-Moderate
*Veronicella sloanii*	Pancake slug	UK	10 = Easy-Moderate
*Myriapoda* sp.	Myriapod	AUS, ESP	10 = Easy-Moderate
*Chilopoda* sp.	Centipede	AUS	15 = Moderate
*Diplopoda* sp.	Millipede	AUS	10 = Easy-Moderate
*Archispirostreptus gigas*	Giant millipede	UK	10 = Easy-Moderate
*Orthoporus ornatus*	Chocolate millipede	UK	10 = Easy-Moderate
*Tonkinbolus dollfusi*	Rainbow millipede	UK	10 = Easy-Moderate
*Macropanesthia rhinoceros*	Burrowing cockroach	AUS	5 = Easy
*Parcoblatta* sp.	Wood cockroaches	AUS	5 = Easy
*Gromphadorhina portentosa*	Hissing cockroach	USA, NL, UK	5 = Easy
*Aphonopelma chalcodes*	Arizona desert tarantula	UK	25 = Difficult
*Brachypelma smithi*	Red-knee tarantula	USA, CAN, UK	25 = Difficult
*Grammostola pulchra*	Brazilian black tarantula	UK	25 = Difficult
*Tliltocatl albopilosus*	Honduran curly-haired tarantula	UK	25 = Difficult
*Tliltocatl albopilosus*	Curly-haired tarantula	USA	25 = Difficult
*Ctenizidae* sp.	Trapdoor spider	AUS	25 = Difficult
*Badumna insignis*	Black house spider	AUS	25 = Difficult
*Sparassidae* sp.	Huntsman spider	AUS	25 = Difficult
*Lycosidae* sp.	Wolf spider	AUS	25 = Difficult
*Lampona* sp.	White-tail spider	AUS	25 = Difficult
*Latrodectus hasselti*	Redback spider	AUS	25 = Difficult
*Eriophora transmarina*	Garden orb weaver spider	AUS	25 = Difficult
*Theraphosa blondi*	Bird-eating spider	AUS	25 = Difficult
*Lasiodora parahybana*	Salmon pink bird eating spider	USA, UK	25 = Difficult
*Selenocosmia* sp.	Australian tarantula	AUS	25 = Difficult
*Grammostola pulchripes*	Golden-knee tarantula	UK	25 = Difficult
*Grammostola rosea*	Red Chile rose tarantula	USA, CAN, NL, UK	25 = Difficult
*Tarantula* sp.	Tarantula	ESP	25 = Difficult
*Scorpiones* sp.	Scorpion	AUS, ESP	25 = Difficult
*Anuroctonus phaiodactylus*	Burrowing scorpion	AUS	25 = Difficult
*Urodacus elongatus*	Flinders Ranges scorpion	AUS	25 = Difficult
*Hadrurus arizonensis*	Desert scorpion	AUS	25 = Difficult
*Hadogenes troglodytes*	Flat rock scorpion	UK	25 = Difficult
*Pandinus imperator*	Emperor scorpion	USA, CAN, UK	25 = Difficult
*Heterometrus* sp.	Forest scorpion	UK	25 = Difficult
*Thelyphonida* sp.	Whip scorpion	UK	20 = Moderate-Difficult
*Amblypygi* sp.	Tailless whip scorpion	USA, UK	20 = Moderate-Difficult
*Mastigoproctus giganteus*	Giant vinegaroon	USA, UK	20 = Moderate-Difficult
*Phasmatodea* sp.	Stick insect	AUS, ESP, UK	10 = Easy-Moderate
*Tropidoderus childrenii*	Children’s stick insect	AUS	10 = Easy-Moderate
*Onchestus rentzi*	Crowned stick insect	AUS	10 = Easy-Moderate
*Phyllium monteithi*	Phylium Monteith stick insect	AUS	10 = Easy-Moderate
*Eurycnema goliath*	Goliath stick insect	AUS	10 = Easy-Moderate
*Peruphasma schultei*	Black velvet stick insect	UK	10 = Easy-Moderate
*Phyllidae* sp.	Leaf insect	USA, ESP, UK	10 = Easy-Moderate
*Extatosoma tiaratum*	Macleays spectre	UK	10 = Easy-Moderate
*Acrophylla titan*	Titan’s stick insect	AUS	10 = Easy-Moderate
*Aretaon asperrimus*	Thorny stick insect	UK	10 = Easy-Moderate
*Hymenopus coronatus*	Flower praying mantis	UK	10 = Easy-Moderate
*Deroplatys* sp.	Dead leaf mantis	UK	10 = Easy-Moderate
*Pachnoda* marginata	Pachnoda fruit beetle	UK	10 = Easy-Moderate
*Grylloidea* sp.	Cricket	AUS	10 = Easy-Moderate
*Tenebrio molitor*	Mealworm	AUS	10 = Easy-Moderate
*Anthophila* sp.	Bees	AUS	10 = Easy-Moderate

Keys: AUS = Australia; USA = United States of America; CAN = Canada; ESP = Spain; NL = The Netherlands; UK = United Kingdom. EMODE assesses species suitability for keeping based on husbandry challenge as ‘easy’, ‘moderate’, ‘difficult’, or ‘extreme’.

**Table 4:** Fishes involved in handling and other practices at mobile zoos by species and country of where used, and their EMODE * ‘suitability to keep’ scores.

Species		Country	EMODE Score/Challenge
Scientific Name	Common Name		
*Amphiprion ocellaris*	Clownfish	USA, UK	25 = Difficult
*Cyprinus carpio*	Carp	UK	10 = Easy-Moderate
*Paracanthurus hepatus*	Blue tang	USA	25 = Difficult
*Rhinecanthus aculeatus*	Clown triggerfish	USA	20 = Moderate-Difficult
*Rhinoptera bonasus*	Cownose stingray	USA	25 = Difficult
*Hypanus americanus*	Southern stingray	USA	25 = Difficult
*Myliobatoidei* sp.	Stingray	AUS	25 = Difficult
*Selachimorpha* sp.	Sharks	AUS	33 = Extreme
*Pomacanthus imperator*	Emperor angelfish	USA	25 = Difficult
*Pterois* sp.	Lion fish	USA	25 = Difficult
*Gymnomuraena zebra*	Zebra moray eel	USA	25 = Difficult
*Diodontidae* sp.	Porcupinefish	AUS	25 = Difficult
*Hippocampus* sp.	Seahorse	AUS	25 = Difficult
*Hippocampus abdominalis*	Pot belly seahorse	AUS	25 = Difficult
*Lactoria cornuta*	Cow fish	AUS	25 = Difficult

**Table 5:** Amphibians involved in handling and other practices at mobile zoos by species and country of where used, and their EMODE ‘suitability to keep’ scores.

Species		Country	EMODE Score/Challenge
Scientific Name	Common Name		
*Rhinella marina*	Marine/cane toad	AUS, UK	23 = Difficult
*Anura* sp.	Frog	AUS	23 = Difficult
*Hylidae* sp.	Tree frog	AUS	23 = Difficult
*Litoria caerulea*	Green tree frog	AUS	23 = Difficult
*Litoria splendida*	Splendid green tree frog	AUS	23 = Difficult
*Bufo bufo*	Common European toad	UK	23 = Difficult
*Incilius alvarius*	Colorado river toad	UK	23 = Difficult
*Pyxicephalus adspersus*	African bullfrog	USA, NL, UK	23 = Difficult
*Ranoidea caerulea*	White’s tree frog	UK	28 = Difficult-Extreme
*Theloderma corticale*	Mossy tree frog	USA, UK	28 = Difficult-Extreme
*Agalychnis callidryas*	Red-eyed tree frog	CAN, UK	28 = Difficult-Extreme
*Polypedates otilophus*	Borneo eared frog	USA	28 = Difficult-Extreme
*Trachycephalus resinifictrix*	Amazonian milk frog	UK	28 = Difficult-Extreme
*Urodela* sp.	Salamanders	AUS	33 = Extreme
*Salamandra salamandra*	Fire salamander	UK	33 = Extreme
*Ambystoma tigrinum*	Tiger salamander	NL, UK	33 = Extreme
*Ambystoma mexicanum*	Axolotl	AUS	23 = Difficult

**Table 6:** Reptiles involved in handling and other practices at mobile zoos by species and country of where used, and their EMODE ‘suitability to keep’ scores.

Species		Country	EMODE Score/Challenge
Scientific Name	Common Name		
Chelonians			
*Glyptemys insculpta*	Wood turtle	UK	23 = Difficult
*Rhinoclemmys pulcherrima*	Wood turtle	ESP	23 = Difficult
*Terrapene carolina*	Box turtle	USA, CAN	23 = Difficult
*Trachemys scripta scripta*	Yellow-bellied turtle	CAN	23 = Difficult
*Geoemyda spengleri*	Black-breasted leaf turtle	USA	23 = Difficult
*Graptemys pseudogeographica kohni*	Mississippi map terrapin	UK	23 = Difficult
*Emydura macquarii*	Macquarie turtle	AUS	23 = Difficult
*Chelodina colliei*	Oblong turtle	AUS	23 = Difficult
*Myuchelys latisternum*	Saw-shelled turtle	AUS	23 = Difficult
*Chelodina longicollis*	Long-necked turtle	AUS	23 = Difficult
*Pelodiscus sinensis*	Soft-shelled turtle	USA	23 = Difficult
*Geochelone elegans*	Star tortoise	UK	23 = Difficult
*Centrochelys sulcata*	Sulcata tortoise	USA, CAN, ESP, UK	33 = Extreme
*Aldabrachelys gigantea*	Alabra giant tortoise	USA	33 = Extreme
*Gopherus agassizii*	Desert tortoise	USA	23 = Difficult
*Kinixys belliana*	Western hinge-back tortoise	UK	23 = Difficult
*Indotestudo elongate*	Elongated tortoise	UK	23 = Difficult
*Chelonoidis denticulatus*	Yellow-footed tortoise	UK	23 = Difficult
*Chelonoidis carbonarius*	Red-footed tortoise	CAN	23 = Difficult
*Astrochelys radiata*	Radiated tortoise	USA	23 = Difficult
*Testudo hermanni*	Hermann’s tortoise	NL, ESP, UK	23 = Difficult
*Testudo horsfieldii*	Horsfield’s tortoise	NL, ESP, UK	23 = Difficult
*Chelonoidis carbonarius*	Red-footed tortoise	ESP, UK	23 = Difficult
*Stigmochelys pardalis*	Leopard tortoise	ESP, UK	23 = Difficult
*Trachemys scripta*	Yellow-bellied terrapin	ESP	23 = Difficult
Crocodiles			
*Crocodylus niloticus*	Nile crocodile	USA, UK	33 = Extreme
*Alligator mississippiensis*	American alligator	USA, CAN	33 = Extreme
*Crocodylidae* sp.	Saltwater and Freshwater crocodile	AUS	33 = Extreme
*Crocodylidae* sp.	Freshwater crocodile	AUS	33 = Extreme
*Paleosuchus palpebrosus*	Cuvier’s dwarf caiman	CAN	33 = Extreme
*Caiman crocodilus*	Spectacled caiman	CAN	33 = Extreme
Lizards			
*Furcifer pardalis*	Panther chameleon	CAN, UK	28 = Difficult-Extreme
*Chamaeleo calyptratus*	Yemen chameleon	ESP, NL	28 = Difficult-Extreme
*Chlamydosaurus kingii*	Frilled-neck lizard	AUS, CAN	28 = Difficult-Extreme
*Ctenophorus nuchalis*	Central netted dragon	AUS	23 = Difficult
*Pogona vitticeps*	Bearded dragon	USA, CAN, ESP, NL, UK	23 = Difficult
*Acanthosaura* sp.	Horned dragon	UK	28 = Difficult-Extreme
*Iguana iguana*	Green iguana	USA, CAN, ESP, UK	28 = Difficult-Extreme
*Physignathus cocincinus*	Water dragon	ESP, UK	28 = Difficult-Extreme
*Hydrosaurus amboinensis*	Sailfin lizard	USA	28 = Difficult-Extreme
*Calotes* sp.	Agama	UK	23 = Difficult
*Uromastyx ornata*	Uromastyx	USA, CAN, UK	23 = Difficult
*Salvator merianae*	Argentinian tegu	USA, CAN, UK	28 = Difficult-Extreme
*Varanus salvator*	Salvator monitor	UK	28 = Difficult-Extreme
*Varanus acanthurus*	Spiny-tailed monitor	UK	28 = Difficult-Extreme
*Varanus bengalensis*	Bengal monitor	UK	28 = Difficult-Extreme
*Varanus exanthematicus*	Savannah monitor	USA, CAN, NL, UK	28 = Difficult-Extreme
*Varanus tristis*	Black-headed monitor	USA	28 = Difficult-Extreme
*Varanus griseus*	Desert monitor	ESP	28 = Difficult-Extreme
*Varanus* sp.	Goanna/monitor lizards	AUS	28 = Difficult-Extreme
*Varanus komodoensis*	Komodo dragon	CAN	33 = Extreme
*Correlophus ciliatus*	Crested gecko	CAN, UK	23 = Difficult
*Eublepharis macularius*	Leopard gecko	USA, CAN, ESP, NL, UK	23 = Difficult
*Rhacodactylus leachianus*	Giant gecko	UK	28 = Difficult-Extreme
*Rhacodactylus auriculatus*	Gargoyle gecko	UK	23 = Difficult
*Underwoodisaurus milii*	Thick-tailed gecko	AUS	23 = Difficult
*Phelsuma m. madagascariensis*	Madagascan day gecko	USA, UK	23 = Difficult
*Nephrurus* sp.	Knob-tailed gecko	AUS, USA	23 = Difficult
*Tribolonotus gracilis*	Crocodile skink	USA, UK	28 = Difficult-Extreme
*Eumeces schneiderii*	Berber skink	NL, UK	23 = Difficult
*Mochlus fernandi*	Fire skink	USA	23 = Difficult
*Egernia stokesii*	Gidgee skink	AUS	23 = Difficult
*Tiliqua multifasciata*	Centralian blue-tongued skink	AUS, CAN	23 = Difficult
*Tiliqua rugosa*	Shingleback lizard	AUS	23 = Difficult
*Tiliqua scincoides*	Melanistic blue-tongued lizard	AUS	23 = Difficult
*Tiliqua gigas*	Blue-tongued skink	AUS, USA, NL, UK	23 = Difficult
*Pseudopus apodus*	Legless lizard	USA	23 = Difficult
*Pygopus schraderi*	Eastern hooded scaly foot lizard	AUS	23 = Difficult
*Moloch horridus*	Moloch	ESP	28 = Difficult-Extreme
*Heloderma suspectum*	Gila monster	CAN	28 = Difficult-Extreme
Snakes			
*Boa constrictor*	Boa constrictor	USA, CAN, NL, UK	28 = Difficult-Extreme
*Boa constrictor*	Red-tailed boa constrictor	CAN	28 = Difficult-Extreme
*Boa constrictor imperiator*	Hog island boa	UK	28 = Difficult-Extreme
*Eryx colubrinus*	Kenyan sand boa	UK	23 = Difficult
*Eryx jaculus*	Sand boa	USA, UK,	23 = Difficult
*Epicrates cenchria*	Rainbow boa	CAN, NL, UK	28 = Difficult
*Lichanura trivirgata*	Rosy boa	NL, UK	23 = Difficult
*Hoplocephalus stephensii*	Stephens’ banded snake	AUS	23 = Difficult
*Python regius*	Ball python	USA, CAN, ESP, NL, UK	23 = Difficult
*Python curtus*	Blood python	USA	28 = Difficult-Extreme
*Python bivittatus*	Burmese python	CAN, UK	28 = Difficult-Extreme
*Antaresia childreni*	Children’s python	CAN, UK	23 = Difficult
*Morelia bredli*	Bredl’s python	AUS	23 = Difficult
*Morelia spilota metcalfei*	Murray Darling python	AUS	23 = Difficult
*Morelia spilota*	Carpet python	AUS, NL	23 = Difficult
*Liasis olivaceus*	Olive python	AUS, CAN	23 = Difficult
*Antaresia maculosa*	Spotted python	UK	23 = Difficult
*Malayopython reticulatus*	Reticulated python	USA, CAN, UK	28 = Difficult-Extreme
*Morelia viridis*	Green tree python	CAN, UK	28 = Difficult-Extreme
*Leiopython albertisii*	D’Albertis’ python	UK	23 = Difficult
*Aspidites ramsayi*	Woma python	AUS, USA	23 = Difficult
*Aspidites melanocephalus*	Black headed python	AUS	23 = Difficult
*Lampropeltis* sp.	Common kingsnake	USA	23 = Difficult
*Lampropeltis californiae*	Californian kingsnake	USA, ESP	23 = Difficult
*Lampropeltis alterna*	Grey-banded kingsnake	UK	23 = Difficult
*Lampropeltis triangulum*	Milk snake	USA, NL, UK	23 = Difficult
*Pantherophis guttatus*	Corn snake	USA, CAN, ESP, UK	23 = Difficult
*Heterodon nasicus*	Weston hognose snake	UK	23 = Difficult
*Euprepiophis mandarinus*	Mandarin rat snake	UK	23 = Difficult
*Erpeton tentaculatum*	Tentacled snake	USA	23 = Difficult
*Hydrodynastes gigas*	False water cobra	UK	23 = Difficult
*Gonyosoma oxycephalum*	Red-tailed green rat snake	USA	23 = Difficult

**Table 7:** Birds involved in handling and other practices at mobile zoos by species and country of where used, and their EMODE ‘suitability to keep’ scores.

Species		Country	EMODE Score/Challenge
Scientific Name	Common Name		
*Tyto alba*	Barn owl	USA, ESP, UK	28 = Difficult
*Ninox boobook*	Boobook owl	UK	28 = Difficult
*Asio otus*	Long-eared owl	ESP, UK	28 = Difficult
*Strix aluco*	Tawny owl	NL, UK	28 = Difficult
*Strigidae* sp.	Screech owl	UK	28 = Difficult
*Athene noctua*	Little owl	ESP, UK	28 = Difficult
*Strix leptogrammica*	Malaysian wood owl	UK	28 = Difficult
*Bubo bubo*	Eurasian eagle owl	ESP	28 = Difficult
*Bubo africanus*	African spotted eagle owl	ESP, UK	28 = Difficult
*Bubo lacteus*	Verreaux’s eagle owl	USA	28 = Difficult
*Bubo scandiacus*	Snowy owl	ESP	28 = Difficult
*Falco peregrinus*	Peregrine falcon	ESP	28 = Difficult
*Aquila nipalensis*	Steppe eagle	ESP	28 = Difficult
*Ptilopsis granti*	Southern white-faced scop owl	UK	28 = Difficult
*Otus scops*	Eurasian scops owl	ESP	28 = Difficult
*Podargus papuensis*	Papuan frogmouth	AUS	28 = Difficult
*Podargus strigoides*	Tawny frogmouth	AUS	28 = Difficult
*Strigiformes* sp.	Owl	AUS	28 = Difficult
*Parabuteo unicinctus*	Harris hawk	ESP, UK	28 = Difficult
*Falco tinnunculus*	Common kestrel	ESP	28 = Difficult
*Falco sparverius*	American kestrel	ESP, UK	28 = Difficult
*Gyps rueppelli*	Ruppel’s griffon vulture	USA	33 = Extreme
*Bycanistes brevis*	Silvery-cheeked hornbill	USA	33 = Extreme
*Rhabdotorrhinus corrugatus*	Wrinkled hornbill	USA	33 = Extreme
*Threskiornis spinicollis*	Straw-necked ibis	USA	28 = Difficult
*Psittacus erithacus*	African grey parrot	USA, NL, UK	33 = Extreme
*Amazona oratrix*	Amazon parrot	USA, UK	33 = Extreme
*Psittaciformes* sp.	Parrot	AUS	33 = Extreme
*Amazona ochrocephala*	Yellow-crowned Amazon	UK	33 = Extreme
*Ara ararauna*	Blue and gold macaw	USA, ESP, NL, UK	33 = Extreme
*Ara macao*	Macaw	AUS	33 = Extreme
*Pionites melanocephalus*	Black-headed caique	UK	33 = Extreme
*Nymphicus hollandicus*	Cockatiel	USA, UK	28 = Difficult-Extreme
*Calyptorhynchus banksii*	Red-tailed black cockatoo	AUS	33 = Extreme
*Cacatuidae* sp.	Cockatoo	AUS	33 = Extreme
*Cacatua alba*	Cockatoo	USA	33 = Extreme
*Pyrrhura molinae*	Conure	USA, UK	33 = Extreme
*Psittacula krameria*	Ring-necked parakeet	USA, UK	28 = Difficult-Extreme
*Trichoglossus rubritorquis*	Red-collared lorikeet	UK	28 = Difficult-Extreme
*Trichoglossus moluccanus*	Rainbow lorikeet	AUS, USA	28 = Difficult-Extreme
*Agapornis* sp.	Love bird	USA	28 = Difficult-Extreme
*Spheniscus demersus*	African black-footed penguin	USA	28 = Difficult-Extreme
*Gymnorhina tibicen*	Australian magpie	USA	28 = Difficult-Extreme
*Pica pica*	Magpie	AUS, ESP	28 = Difficult-Extreme
*Corvus* sp.	Crow/raven	AUS, ESP	28 = Difficult-Extreme
*Entomyzon cyanotis*	Blue-faced honeyeater	USA	28 = Difficult-Extreme
*Lophotis gindiana*	Buff-crested bustard	USA	28 = Difficult-Extreme
*Pelecanus onocrotalus*	Great white pelican	USA	28 = Difficult-Extreme
*Grus carunculate*	Wattled crane	USA	28 = Difficult-Extreme
*Leptoptilos crumenifer*	Marabou stork	USA	28 = Difficult-Extreme
*Ciconia Ciconia*	White stork	USA	28 = Difficult-Extreme
*Vanellus miles*	Masked lapwing	USA	28 = Difficult-Extreme
*Casuarius* sp.	Cassowaries	AUS	33 = Extreme
*Dromaius novaehollandia*	Emu	AUS	33 = Extreme
*Struthio* sp.	Ostrich	USA, NL	33 = Extreme
*Pavo cristatus*	Peafowl	USA	28 = Difficult-Extreme
*Garrulax leucolophus*	White-crested laughing thrush	USA	28 = Difficult-Extreme
*Dacelo* sp.	Kookaburra	AUS	28 = Difficult-Extreme
*Columba livia domestica*	Pigeon	USA	10 = Easy-Moderate
*Gallus gallus domesticus*	Chicken	USA, CAN, ESP, UK	10 = Easy-Moderate
*Meleagris* sp.	Turkey	AUS	10 = Easy-Moderate
*Anas platyrhynchos domesticus*	Call duck	USA, ESP, NL, UK	15 = Moderate
*Anatidae* sp.	Duck	AUS, CAN, ESP	15 = Moderate

**Table 8:** Mammals involved in handling and other practices at mobile zoos by species and country of where used, and their EMODE ‘suitability to keep’ scores.

Species		Country	EMODE Score/Challenge
Scientific Name	Common Name		
*Ateles* sp.	Spider monkey	USA	28 = Difficult-Extreme
*Aotus* sp.	Owl monkey	USA	28 = Difficult-Extreme
*Cebinae* sp.	Capuchin monkey	USA	28 = Difficult-Extreme
*Macaca* sp.	Macaque	AUS	28 = Difficult-Extreme
*Callithrix jacchus*	Marmoset	AUS	28 = Difficult-Extreme
*Varecia rubra*	Red-ruffed lemur	USA	28 = Difficult-Extreme
*Arctictis binturong*	Bearcat	USA	28 = Difficult-Extreme
*Prionailurus bengalensis*	Leopard cat	USA	28 = Difficult-Extreme
*Meles meles*	European badger	ESP	28 = Difficult-Extreme
*Melogale personata*	Burmese badger	USA	28 = Difficult-Extreme
*Potos* sp.	Kinkajou	USA, ESP	28 = Difficult-Extreme
*Tamandua* sp.	Anteater	USA	28 = Difficult-Extreme
*Coendou* sp.	Porcupine	USA	28 = Difficult-Extreme
*Erethizon* sp.	Porcupine	ESP	28 = Difficult-Extreme
*Tolypeutes* sp.	Armadillo	USA, UK	33 = Extreme
*Nasua* sp.	Coatimundi	USA, ESP, UK	33 = Extreme
*Genette genetta*	Genet	ESP	28 = Difficult-Extreme
*Suricata suricatta*	Meerkat	UK	33 = Extreme
*Bradypus* sp.	Sloth	USA	33 = Extreme
*Mephitis* sp.	Black and white skunk	USA, ESP, UK	23 = Difficult
*Procyon* sp.	Raccoon	USA	28 = Difficult-Extreme
*Lutrinae* sp.	Otter	USA	28 = Difficult-Extreme
*Hydrochoerus hydrochaeris*	Capybara	USA	28 = Difficult-Extreme
*Marmota monax*	Groundhog	USA	23 = Difficult
*Didelphis* sp.	Opossum	UK	23 = Difficult
*Trichosurus vulpecula*	Brush-tailed possum	AUS	23 = Difficult
*Burramys parvus*	Mountain pygmy possum	AUS	23 = Difficult
*Mungos mungo*	Banded mongoose	USA	28 = Difficult-Extreme
*Dolichotis patagonum*	Patagonian mara	USA, UK	28 = Difficult
*Cricetomys gambianus*	Gambian pouched rat	USA, NL, UK	23 = Difficult
*Chinchilla* sp.	Chinchilla	USA, CAN, ESP, NL, UK	25 = Difficult
*Pachyuromys duprasi*	Duprasi	UK	15 = Moderate
*Cynomys* sp.	Prairie dog	USA, UK	28 = Difficult-Extreme
*Petaurus breviceps*	Sugar glider	AUS, USA, CAN, UK	33 = Extreme
*Octodon degus*	Degu	USA, UK	25 = Difficult
*Sciuridae* sp.	Chipmunk	USA, UK	23 = Difficult
*Atelerix algirus*	African pygmy hedgehog	USA, CAN, ESP, UK	28 = Difficult-Extreme
*Erinaceus* sp.	Hedgehog	USA	28 = Difficult-Extreme
*Lepus arcticus*	Arctic hare	USA	23 = Difficult
*Oryctolagus cuniculus*	Rabbit	AUS, USA, CAN, ESP, NL, UK,	15 = Moderate
*Oryctolagus cuniculus domesticus*	Dwarf rabbit	USA, UK	20 = Moderate-Difficult
*Cavia porcellus*	Guinea pig	AUS, USA, CAN, ESP, NL, UK	10 = Easy-Moderate
*Mesocricetus auratus*	Hamster	CAN, NL	15 = Moderate
*Rattus norvegicus domestica*	Rat	USA, NL, UK	10 = Easy-Moderate
*Mus musculus*	Mouse	AUS, NL, UK	10 = Easy-Moderate
*Mustela furo*	Ferret	AUS, USA, CAN, ESP, UK	15 = Moderate
*Hemicentetes* sp.	Tenrec	UK	28 = Difficult-Extreme
*Vulpes* sp.	Fox	UK, ESP	23 = Difficult
*Otocyon megalotis*	Bat-eared fox	USA	23 = Difficult
*Felis catus*	Cat	NL, ESP	10 = Easy-Moderate
*Canis familiaris*	Dog	AUS, USA, ESP, NL, UK	10 = Easy-Moderate
*Canis dingo*	Dingo	AUS	10 = Easy-Moderate
*Sus scrofa domesticus*	Pig	AUS, USA, NL, UK	15 = Moderate
*Sus domesticus*	Pot-bellied pig	CAN	15 = Moderate
*Capra* sp.	Goat	AUS, USA, CAN, ESP, NL	15 = Moderate
*Ovis aries*	Sheep	AUS, USA, CAN, ESP, NL	15 = Moderate
*Bos taurus*	Cow	AUS, USA, NL	15 = Moderate
*Equus zebra*	Zebra	USA	15 = Moderate
*Equus ferus caballus*	Horse	AUS, USA, CAN, NL	15 = Moderate
*Equus* *africanus asinus*	Donkey	AUS, USA, CAN	15 = Moderate
*Camelus* sp.	Camel	AUS, USA, NL	15 = Moderate
*Vicugna pacos*	Alpaca	AUS, USA, CAN, NL	15 = Moderate
*Lama glama*	Llama	AUS	15 = Moderate
*Vombatidae* sp.	Wombat	AUS	28 = Difficult-Extreme
*Tachyglossidae* sp.	Echidna	AUS	28 = Difficult-Extreme
*Sminthopsis crassicaudata*	Fat-tailed dunnart	AUS	23 = Difficult
*Sarcophilus harrisii*	Potoroo	AUS	28 = Difficult-Extreme
*Phascolarctos cinereus*	Koala	AUS	28 = Difficult-Extreme
*Macropodidae* sp.	Kangaroo/wallaby	AUS, USA, ESP	23 = Difficult
*Dasyurus maculatus*	Tiger quoll	AUS	23 = Difficult
*Dasyurus viverrinus*	Eastern quoll	AUS	23 = Difficult
*Cervidae* sp.	Deer	AUS	15 = Moderate
*Bubalus* sp.	Buffalo	AUS	23 = Difficult
*Bettongia* sp.	Bettong	AUS	23 = Difficult
*Bettongia penicillata*	Brush-tailed bettong	AUS	23 = Difficult
*Aepyprymnus rufescens*	Rufus bettong	AUS	23 = Difficult

**Table 9:** Examples of common educational messaging (anonymised) associated with mobile zoos, and critical comments.

Claim	Critical Comment	Example References Supporting Critical Comments
*‘Many captive-bred reptiles are now domesticated.’*	False. There are no domesticated species or types of reptiles.	[6,104,105,106,107]
*‘Most invertebrates, amphibians, and reptiles are low maintenance and easy to keep as pets.’*	False. Strong innate behavioural drive states, highly specific environmental cues and needs, and relative lack of biological information infer comparatively high husbandry challenges.	[51,104,105,108,109,110,111,112,113]
*‘Invertebrates, amphibians, and reptiles need little mental stimulation or space.’*	Misleading. Many if not most relevant species are well-documented to naturally occupy large home ranges, and prefer greater space in captive settings.	[104,105,108,114,115,116,117,118] (See also 4.3.2. ‘Biological considerations, needs, & preferences’)
*‘Invertebrates, fishes, amphibians, and reptiles rarely show signs of stress.’*	False. Deficits in proper observation and knowledge bases result in animal behaviours being under-investigated for stress.	[37,104,105,108,110,119,120]
*‘If animals were stressed by handling they would not eat, grow or breed.’*	Misleading. Positive appetite, growth, and reproduction are unreliable indicators of quiescence or absence of stress. Animals may perceive their handlers as predators.	[104,121,122,123,124] (See also 4.3.2. ‘Biological considerations, needs, & preferences’)
*‘Handwashing prevents contracting salmonellosis and other zoonotic diseases.’*	Misleading. Although helpful in reducing contamination, handwashing does not eliminate all germs or guarantee protection against infection.	[125,126,127,128] (See also 4.3.2. ‘Biological considerations, needs, & preferences’)
*‘Furless and featherless animals, such as reptiles, are especially safe for handling by people with allergies.’*	False. Furless and featherless animals harbour many potential allergens, such as enzymes and excretions that are capable of causing allergic reactions.	[129,130,131,132,133,134,135]
*‘Handling tamed exotic animals is safe.’*	Misleading. Innate ancestral defensive and aggressive psychological and behavioural traits remain even in multigenerational captive-bred and trained animals, regardless of species.	[113,136,137,138,139] (‘See also 4.3.2. ‘Biological considerations, needs, & preferences’)

**Table 10:** Animal welfare concerns identified in peer-reviewed literature that are relevant to mobile zoo practices.

Animal Welfare Concerns	Example References
Frequent handling.	[38,140,141,142,143]
Handling by naïve or novel persons.	[38,140,141,142,143]
Cross-handling of predatory and prey species and associated chemical cue transfer.	[144,145,146]
Use of non-domesticated (wild) species unsuitable for captivity.	[110,147]
Invasive vibrational disturbances.	[114,148,149,150,151,152]
Invasive audio disturbances.	[149,150,153,154,155]
Invasive light disturbances.	[114,149,152,154,156]
Transport stress (often repeated).	[37,39,152,154,157,158]
Lack of voluntary feeding or drinking.	[114,152]
Disturbance of nocturnal species.	[114,152,159]
Poor knowledge of species biological and husbandry needs among handlers and carers.	[106,110]
Subnormal housing and husbandry, display and handling.	[114,147,152]
Poor housing and husbandry (temperature, lighting, humidity, space) conditions at permanent or temporary holding sites.	[37,147,152,160]
Dissemination of emerging infectious diseases to other animals.	[161,162,163,164,165,166]

## Data Availability

Not applicable.
